# Carnivore Use of Avocado Orchards across an Agricultural-Wildland Gradient

**DOI:** 10.1371/journal.pone.0068025

**Published:** 2013-07-02

**Authors:** Theresa M. Nogeire, Frank W. Davis, Jennifer M. Duggan, Kevin R. Crooks, Erin E. Boydston

**Affiliations:** 1 School of Environmental and Forest Sciences, University of Washington, Seattle, Washington United States of America; 2 Bren School of Environmental Science and Management, University of California Santa Barbara, Santa Barbara, California, United States of America; 3 Department of Fish, Wildlife, and Conservation Biology, Colorado State University, Fort Collins, Colorado, United States of America; 4 U.S. Geological Survey, Western Ecological Research Center, Thousand Oaks, California, United States of America; University of Alberta, Canada

## Abstract

Wide-ranging species cannot persist in reserves alone. Consequently, there is growing interest in the conservation value of agricultural lands that separate or buffer natural areas. The value of agricultural lands for wildlife habitat and connectivity varies as a function of the crop type and landscape context, and quantifying these differences will improve our ability to manage these lands more effectively for animals. In southern California, many species are present in avocado orchards, including mammalian carnivores. We examined occupancy of avocado orchards by mammalian carnivores across agricultural-wildland gradients in southern California with motion-activated cameras. More carnivore species were detected with cameras in orchards than in wildland sites, and for bobcats and gray foxes, orchards were associated with higher occupancy rates. Our results demonstrate that agricultural lands have potential to contribute to conservation by providing habitat or facilitating landscape connectivity.

## Introduction

Land-use change is a leading driver of loss of biological diversity globally [1]. As pressures from habitat loss increase, there is growing interest in agricultural landscapes as potential habitat or movement areas for wildlife. Agricultural landscapes are potentially rich in structure, food, and cover, and many native species forage and reproduce in these landscapes [2]. These lands can support moderate diversity of birds, mammals, arthropods, and plants, depending on the intensity of agriculture [3], [4] and on configuration of natural land cover [5].

Mammalian carnivores are frequent targets of conservation efforts [6], and they play a key role in food webs, for example via mesopredator release [7] or trophic downgrading [8]. Because carnivores are typically wide-ranging, it is especially important to consider agricultural landscapes as well as protected areas when forming conservation plans for these species. Wildlife managers and conservation planners currently have little knowledge of carnivore use of agricultural landscapes, but this subject will become increasingly important as agricultural systems continue to expand and protected areas become more isolated. Connectivity between habitat patches is especially critical in human-dominated landscapes [9], but most connectivity models focus on natural vegetation types, not on differences between human-dominated land cover types within such landscapes [10]. When evaluating landscape connectivity for large carnivores, conservation planners have often relied on expert opinion and considered all agriculture as having uniformly low connectivity value (for example [11]–[13]).

Many members of the order Carnivora are omnivorous and feed, in part, on anthropogenic food sources [14]–[16]. Scat analysis has identified cultivated fruit in the diets of carnivores, particularly foxes and stone martens [15]–[17], and Borchert et al. [18] found that at least one orchard type – avocado – was regularly used by carnivores in California. California is a major producer of avocados, with 23,500 hectares of orchards [19] spread across five southern counties. Because avocados grow well on steep slopes, they are planted in a variety of landscape contexts, including hillslopes adjacent to native vegetation as well as valley bottoms adjacent to other types of crops.

We examined the use of avocado orchards by mammalian carnivores across agricultural-wildland gradients in southern California. We assessed whether occupancy of carnivores at motion-activated camera stations was a function of surrounding land cover, and in particular, whether area of orchards influenced carnivore occupancy. If orchards constitute poor quality carnivore habitat relative to natural areas, we would expect to observe carnivores less frequently in orchards than in nearby wildlands.

## Methods

### Study Area

Coastal southern California is highly urbanized and contains about two thirds of California’s 38 million residents; it also has relatively little remaining undeveloped land [20], yet is experiencing rapid population growth [21]. This region has a Mediterranean-type climate, and the dominant natural vegetation types are oak woodland, riparian woodland, sage scrub, and annual grassland. Eleven native members of the order Carnivora occur in this region: American badger (*Taxidea taxus)*, American black bear (*Ursus americanus)*, bobcat (*Lynx rufus*), coyote (*Canis latrans*), gray fox (*Urocyon cinereoargenteus)*, long-tailed weasel (*Mustela frenata*), mountain lion (*Puma concolor)*, raccoon (*Procyon lotor)*, ringtail (*Bassariscus astutus)*, striped skunk (*Mephitis mephitis)*, and Western spotted skunk (*Spilogale gracilis)*.

**Figure 1:**
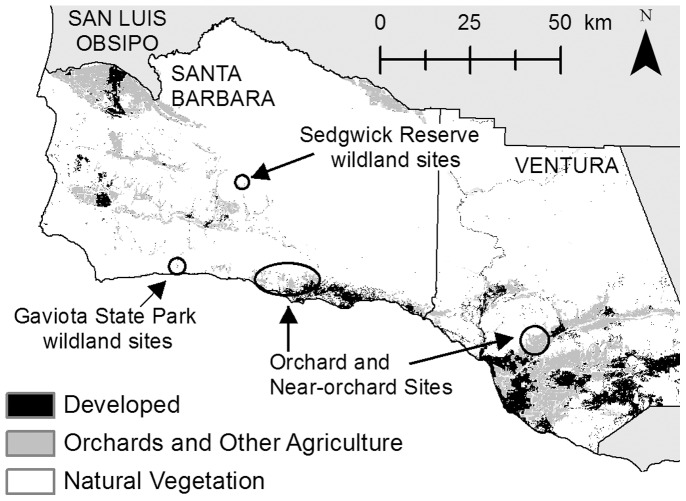
Study area in southern California. Study sites within Santa Barbara and Ventura counties.

**Figure 2:**
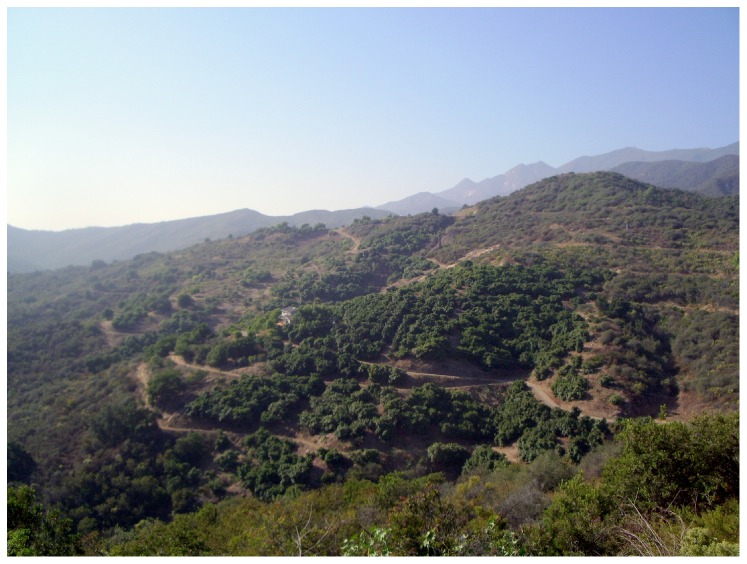
Orchard on hillslope. Typical landscape pattern of steep hills with orchards surrounded by wildlands.

Our study area included avocado orchards and wildlands in Santa Barbara and Ventura counties selected for their position in the landscape and for landowner cooperation (Figure 1). Avocado orchards (hereafter “orchards”) grew on diverse topographies, from steep mountains to flat floodplains, and were surrounded by natural vegetation including sage scrub (Figure 2), oak woodland, grassland vegetation, other agriculture, or low-density development. Wildland sites with only natural vegetation were located at the University of California’s Sedgwick Reserve and Gaviota State Park.

### Land-cover Classification

No single existing land cover map met our habitat mapping needs in terms of scale, accuracy, and legend. We therefore created land cover maps from the National Land Cover Database (NLCD) [22], 2005 Southern California Association of Governments (SCAG), California Department of Water Resources, and the California Avocado Commission. Avocado orchards were identified by the California Avocado Association data, along with the SCAG and Department of Water Resources data. We used NLCD land cover to classify natural habitat types, which were not identified in the SCAG layer. Lands in classes which were essentially open space with substantial human activity (e.g., school yards, golf courses, dirt roads, urban parks, low-density development or developed open space) and which had less than 10% impervious surface were classified as ‘disturbed’. When land-cover layers from the different sources were inconsistent, we verified classifications with ground visits or visual inspection of air photos (National Agriculture Imagery Program 2005, 1 m natural color).

### Camera Stations

We used motion-activated digital cameras (Stealthcam, LLC, Grand Prairie, TX) at 38 sites to detect carnivore species from April 2007 to June 2008, resulting in 1,130 trap nights. Cameras were placed in and around 6 orchards (22 sites in orchards and 6 sites in natural vegetation adjacent to orchards) and 2 continuous wildlands (10 sites), with distance to nearest camera between 30–900 meters (mean = 193 meters).

At all sites, cameras were placed along similar-sized dirt roads, near signs of carnivore activity (e.g., scat) or at trail junctions when possible. We placed scent lure (Pred-a-Getter, Murray’s Lures and Trapping Supplies, Walker, West Virginia) in front of the camera to encourage animals to approach the camera and to stop long enough to be photographed. For each carnivore species at each camera site, we tallied the number of nights in which the species was detected at least once. We considered each 24-hour trap night to begin at 6∶30 am, and cameras were active continuously between 12 and 76 nights (average = 33 nights) at a particular site.

### Detections and Occupancy

To determine the difference in carnivore species richness between land cover types, we examined whether the number of native carnivore species differed among cameras situated in orchards, natural vegetation adjacent to orchards, and continuous wildlands using a likelihood-ratio chi-squared test. We also tested for differences between pairs of land-cover categories with a post-hoc Tukey’s Honest Significant Differences test.

We assessed the influence of landscape variables on carnivore presence at camera stations using a model selection framework to compare occupancy models. We used program PRESENCE v4.6 (PRESENCE, accessed 8/2/12, http://www.mbr-pwrc.usgs.gov/software/presence.html) to estimate occupancy (ψ) and detection rate (p, the probability of detecting a species if it is present) at each camera site [23] for bobcats, coyotes, and gray foxes (species with sufficient detections to permit analyses). This program uses a likelihood approach and has been used with camera-trap data [24], [25], incorporating the effect of both site covariates and sample design. We considered each trap night as a survey. We used Akaike’s Information Criterion corrected for small sample size (AICc) to choose the best-performing models [26].

We began the modeling process by selecting the best detection model for each species while holding occupancy rate constant, as in Negrões et al. [25] and Duggan et al. [27]. We expected that season (wet, November – March [28], versus dry) and land cover at the camera site (orchard, natural vegetation adjacent to orchards, or continuous wildland) could affect detection rate (in addition to occupancy) so we included both as covariates in detection models. Detection covariates, as well as predictors of occupancy described below, were standardized by z-score as described in Donovan and Hines [29].

Next, to determine if spatial clustering affected occupancy, and at what scale, we compared models including site (individual orchards at least 1 km from the perimeter of the nearest neighbor), meta-site (2–3 orchards 3–4 km from one another), or county (Ventura versus Santa Barbara) as predictors of occupancy while including any detection variables selected in the previous step. We then included the covariate from the top-ranked model of spatial scale as a predictor in the candidate model set for occupancy of that species.

Finally, for each species we modeled occupancy while including detection covariates from the top-ranked detection model for that species. Potential predictors of occupancy included land cover at the camera site, distance from each camera to the perimeter of continuous wildland (natural areas contiguous with Gaviota State Park, Los Padres National Forest or adjacent wildlands, ranging from 0–3.4 km), and season (wet versus dry). We also evaluated the degree to which area of orchards and other landscape variables in the neighborhood of a camera influenced carnivore occupancy. To do so, we used the land-cover map to quantify the extent (km^2^) of orchards and covariates (disturbed, shrub/scrub, grassland/herbaceous, and woodland) within a 1,935 m-diameter circle centered on each camera, approximately the average size of a bobcat home range in this region and intermediate between range sizes of foxes and coyotes [30].

We had 38 sites, and therefore examined only single- and double-factor occupancy models to avoid overparameterization. We included all single- and double-factor models in our candidate model set and then conducted model averaging. We report results for the average model, but also include summaries of the top-ranked model and all models within 2 AICc points of this model, indicating substantial support [26]. To compare the selection support for each predictor variable, we also calculated variable importance weights, which are the sum of the model weights of all models that contain a given variable [26]. Averaged models include only models within 2 AICc points of the best model. Variable importance rates are assessed across all models and therefore each variable has equal representation.

## Results

### Camera Stations

Cameras were active for a total of 667 trap nights in orchards, 201 in natural vegetation near orchards, and 262 in wildlands. We detected 8 of the 11 native carnivore species in the study region. Seven native species were detected in orchards: coyote (38 detections), striped skunk (28), bobcat (25), gray fox (20), mountain lion (3), black bear (2), and raccoon (2). Eight native species were detected in natural vegetation: coyote (25), bobcat (21), mountain lion (4), gray fox (7), raccoon (2), badger (1), black bear (1), and striped skunk (1). The 3 native carnivore species not detected included ringtail, spotted skunk, and long-tailed weasel.

### Detections and Occupancy

On average, the number of native species detected per site differed among land-cover classes (χ^2^ = 6.69, df = 2, p = 0.035), but differences between individual classes were not statistically significant (Tukey’s HSD, all p>0.12). The number of native species detected was greatest in orchards (mean = 2.1, SE = 0.36), intermediate in sites with natural vegetation adjacent to orchards (mean = 1.8, SE = 0.48), and lowest in wildland sites (mean = 0.8, SE = 0.40).

The top-ranked detection rate models for coyote and gray fox included land cover at the camera station location (Tables 1, 2), with higher detections in avocado orchards (top model: β = 2.62, SE = 1.09 for coyote and β = 2.21, SE = 1.10 for fox) or near avocado orchard (β = 3.19, SE = 1.10 for coyote and β = 1.47, SE = 1.16 for fox) relative to wildlands. Season was also included in the top-ranked models; for fox, the direction of the effect could not be distinguished from 0 (β = 1.02; SE = 1.16), while for coyote, detection rate was lower in the dry season than in the wet season (β = −0.78, SE = 0.29). These detection covariates were included in all subsequent models. For bobcat, the intercept-only model was the top model, so subsequent bobcat models did not include detection covariates. For all three species, the intercept-only occupancy model was the top-ranked model for spatial variation, thus we did not include spatial variables as predictors of occupancy in our final set of candidate models.

**Table 1:** Top-ranked models of site occupancy (ψ) and detection rate (p) for gray fox.

Model I.D.	K	−2*log-likelihood	ΔAICc	Relative likelihood	*ω*	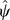 (SE)	p∧ (SE)
Detection (p) ∼							
Land cover + season	5	230	0.00	1.00	0.45	0.47 (0.13)	0.018 (0.025)
Land cover	4	234	0.55	0.76	0.34	0.41 (0.11)	0.017 (0.033)
Season	3	238	2.89	0.24	0.11	0.45 (0.13)	0.015 (0.029)
Intercept only	2	241	2.98	0.23	0.10	0.38 (0.10)	0.059 (0.012)
Occupancy (**ψ**) ∼							
Distwild	6	227	0	1.00	0.14	0.47 (0.14)	0.009 (0.010)
Distwild + woodland	7	224	0.40	0.82	0.11	0.61 (0.09)	0.066 (0.019)
Intercept only	5	230	0.67	0.72	0.10	0.47 (0.13)	0.067 (0.020)
Distwild + Avocado orchard	7	225	0.98	0.61	0.08	0.39 (0.17)	0.068 (0.021)
Shrub	6	228	1.33	0.51	0.07	0.56 (0.12)	0.066 (0.019)
Distwild + disturbed	7	225	1.51	0.47	0.06	0.55 (0.14)	0.065 (0.018)
*Averaged model*					*0.50*	*0.51 (0.13)*	*0.049 (0.016)*

Footnote: All models with **Δ**AICc <2.0, plus the intercept-only models, are reported. K is the number of parameters, **Δ**AICc is the difference between the AICc of the model and the lowest-AICc model, *ω* is the AICc model weight (summed for the averaged model), ψ is the predicted occupancy at a site and *p* is the probability of detecting the species at a given site. Covariate abbreviations: distwild is distance to continuous wildland, land cover is land cover (avocado orchard, near orchard, or wildland) at the camera site, and woodland, avocado orchard, shrub and disturbed refer to the area of that land cover in the neighborhood of the camera site.

**Table 2:** Top-ranked models of site occupancy (ψ) and detection rate (p) for coyote.

Model I.D.	K	−2*log-likelihood	ΔAICc	Relative likelihood	*ω*	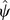 (SE)	p∧ (SE)
Detection (p) ∼							
Land cover+season	5	440	0.00	1.00	0.86	0.68 (0.10)	0.079 (0.019)
Land cover	4	447	4.12	0.13	0.11	0.71 (0.10)	0.069 (0.014)
Season	3	452	6.93	0.03	0.03	0.55 (0.095)	0.10 (0.019)
Intercept only	2	486	38.70	0.00	0.00	0.44 (0.10)	0.056 (0.0068)
Occupancy (**ψ**) ∼							
Disturbed	6	433	0.00	1.00	0.18	0.76 (0.08)	0.056 (0.012)
Grass/herbaceous	6	434	0.68	0.71	0.13	0.75 (0.10)	0.055 (0.012)
Avocado orchard + disturbed	7	432	1.24	0.54	0.10	0.77 (0.10)	0.055 (0.012)
Land Cover + grass/herbaceous	8	429	1.42	0.49	0.09	0.52 (0.10)	0.070 (0.027)
Intercept only	5	440	3.74	0.15	0.03	0.68 (0.10)	0.055 (0.013)
*Averaged model*					*0.53*	*0.72 (0.09)*	*0.06 (0.015)*

Footnote: All models with **Δ**AICc <2.0, plus the intercept-only models, are reported. K is the number of parameters, **Δ**AICc is the difference between the AICc of the model and the lowest-AICc model, *ω* is the AICc model weight (summed for the averaged model), ψ is the predicted occupancy at a site and *p* is the probability of detecting the species at a given site. Covariate abbreviations: distwild is distance to continuous wildland, land cover is land cover (avocado orchard, near orchard, or wildland) at the camera site, and grass/herbaceous, avocado orchard and disturbed refer to the area of that land cover in the neighborhood of the camera site.

Avocado orchards, either at the camera site or in the neighborhood of the camera, were included in at least one competitive occupancy model for all three carnivore species (Tables 1, 2, 3). The area of avocado orchard in the neighborhood of a camera was the most important predictor of bobcat occupancy (model average: β = 0.56, SE = 0.32; Table 4) and was included in all top four models for bobcat occupancy (Table 3). The area of avocado orchards in the neighborhood of a camera was the third most important predictor for gray fox occupancy (β = 0.17, SE = 0.14). For coyote, the area of orchard in the neighborhood had a weak negative effect (β = −0.25, SE = 0.20), but both avocado orchard and ‘near orchard’ at the camera site had a positive effect (avocado orchard: β = 13.41, SE = 6.73; near orchard: β = 13.23, SE = 6.67). Land cover at the camera site was not included in any competitive bobcat or gray fox occupancy models. Distance to continuous wildland was the most important variable for predicting gray fox occupancy (model average: β = −1.16, SE = 0.93) and third most important variable for bobcats (β = −0.16, SE = 0.14; Table 4), with occupancy increasing closer to or within wildland habitat. Distance to continuous wildland was not, however, included in any competitive coyote models. The area of disturbed land in the neighborhood of the camera was included in competitive models for both coyote (β = 4.81, SE = 2.20) and gray fox (β = 0.31, SE = 0.39) occupancy (Tables 1, 2), but large standard error values for fox occupancy suggested a weak influence. Disturbed land was not included in models for bobcat occupancy (Table 3). Woodland, shrub, and grassland/herbaceous vegetation in the neighborhood of a camera had a positive effect on occupancy in all models for all species, except that woodland had a negative effect on gray fox occupancy.

**Table 3:** Top-ranked models of site occupancy (ψ) and detection rate (p) for bobcat.

Model I.D.	K	-2*log-likelihood	ΔAICc	Relative likelihood	*ω*	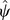 (SE)	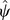 (SE)
Detection (p) ∼							
Intercept only	2	368	0	1.00	0.48	0.44 (0.10)	0.080 (0.012)
Season	3	367	1.26	0.53	0.26	0.52 (0.14)	0.066 (0.019)
Land cover	4	365	2.10	0.35	0.17	0.47 (0.11)	0.071 (0.019)
Land cover+season	5	364	3.27	0.20	0.09	0.55 (0.14)	0.060 (0.022)
Occupancy (**ψ**) ∼							
Avocado orchard + grass/herbaceous	4	363	0	1.00	0.13	0.44 (0.15)	0.081 (0.012)
Avocado orchard + woodland	4	363	0.17	0.92	0.12	0.26 (0.17)	0.081 (0.012)
Avocado orchard	3	365	0.20	0.90	0.12	0.42 (0.13)	0.081 (0.012)
Avocado orchard + distwild	4	363	0.29	0.87	0.11	0.42 (0.15)	0.081 (0.012)
Intercept only	2	368	0.34	0.84	0.11	0.44 (0.10)	0.080 (0.012)
Woodland	3	366	1.11	0.57	0.07	0.28 (0.16)	0.080 (0.012)
Woodland + grass/herbaceous	4	365	1.88	0.39	0.05	0.35 (0.15)	0.079 (0.012)
*Averaged model*					*0.71*	*0.38 (0.14)*	*0.080 (0.012)*

Footnote: All models with **Δ**AICc <2.0, plus the intercept-only models, are reported. K is the number of parameters, **Δ**AICc is the difference between the AICc of the model and the lowest-AICc model, *ω* is the AICc model weight (summed for the averaged model), ψ is the predicted occupancy at a site and *p* is the probability of detecting the species at a given site. Covariate abbreviations: distwild is distance to continuous wildland, land cover is land cover (avocado orchard, near orchard, or wildland) at the camera site, and woodland, grass/herbaceous, shrub, avocado orchard and disturbed refer to the area of that land cover in the neighborhood of the camera site.

**Table 4:** Variable importance weights (*ω*) for predictors of occupancy for bobcats, coyotes, and gray foxes.

Bobcat		Coyote		Gray fox	
Covariate	∑ *ω*	Covariate	∑ *ω*	Covariate	∑ *ω*
Avocado orchard	0.58	Disturbed	0.50	Distance to wildland	0.51
Woodland	0.29	Grass/herbaceous	0.47	Shrub	0.21
Grass/herbaceous	0.28	Avocado orchard	0.15	Avocado orchard	0.18
Distance to wildland	0.13	Shrub	0.12	Woodland	0.18
Season	0.09	Land cover at site	0.10	Disturbed	0.13
Shrub	0.07	Woodland	0.10	Grass/herbaceous	0.11
Disturbed	0.06	Season	0.08	Season	0.09
Land cover at site	0.03	Distance to wildland	0.06	Land cover at site	0.05

## Discussion

Carnivores were detected with surprising frequency in avocado orchards. We detected most carnivore species native to coastal southern California in avocado orchards, and these orchards were used frequently by bobcats, coyotes and gray foxes. Further, we detected more carnivore species in orchards than in wildland sites. Although orchards are often adjacent to wildlands, the presence of carnivores in orchards does not appear to be simply an artifact of landscape context. If this were the case, we would expect to find more carnivores in wildlands than in orchards, which we did not. We would also expect to find that distance to continuous wildland was a more consistently important predictor in our models; although it was the strongest predictor of occupancy for gray fox, it was present in only one competitive model for bobcat occupancy and no competitive models for coyote.

The food subsidy value of avocados may explain why omnivores such as bears, coyotes, and raccoons were present in orchards. Indeed, remote cameras have recorded these species eating avocados in southern California (M. Borchert, U.S. Forest Service, personal communication), but why obligate carnivores like mountain lions and bobcats would be present in orchards is less clear. Orchards may provide good cover for carnivores; many of these species are habitat generalists, and orchards often replace oak woodlands with structurally similar vegetation. Irrigation lines in orchards act as a rare source of perennial water in arid landscapes. In our study, we did not find an effect of wet versus dry season on occupancy, as might be expected if carnivores were attracted by water sources. However, irrigation lines, combined with abundant avocados, might simulate year-round wet-season conditions for small mammals, perhaps leading to bottom-up effects in these agricultural systems. Future research could assess whether orchards are providing more food and water for small mammals than native vegetation, and whether a relative increase in prey might help explain the use of these lands by carnivores [31]. Finally, further study could evaluate whether the presence of infrequently-used dirt roads in orchards might appeal to animals moving across densely vegetated landscapes.

There is growing interest in managing for movement of wild animals through agricultural areas [10], [32]. Knowing the value of different land-cover types for habitat or movement can inform conservation decisions regarding which lands should be purchased or put under easements, or which areas are most suitable for the placement of highway crossings [12]. Avocado orchards appear to serve as both foraging and movement habitat for most carnivore species in California, and conservation easements or other incentives to keep land in orchards could offer a cost-effective conservation strategy. Such alternative conservation strategies are particularly important when considering agriculture (including avocados) in Mediterranean-type ecosystems, which are highly threatened [33].
